# Spin–Orbit Torque Booster in an Antiferromagnet via Facilitating a Global Antiferromagnetic Order: A Route toward an Energy-Efficient Memory

**DOI:** 10.1021/acsami.4c15453

**Published:** 2024-11-14

**Authors:** Hao-Kai Chang, Kuan-Yu Chi, Yu-Lon Lin, Yu-Hsien Lai, Yen-Lin Huang, Chi-Feng Pai, Chao-Yao Yang

**Affiliations:** † Department of Materials Science and Engineering, National Yang Ming Chiao Tung University, Hsinchu 300093, Taiwan; ‡ Department of Materials Science and Engineering, Center of Atomic Initiative for New Materials, and Center for Quantum Science and Engineering, National Taiwan University, Taipei 10617, Taiwan; § Center for Emergent Functional Matter Science, National Yang Ming Chiao Tung University, Hsinchu 300093, Taiwan

**Keywords:** antiferromagnet, Néel order, spin−orbit torque, X-ray magnetic linear dichroism, magnon, easy plane

## Abstract

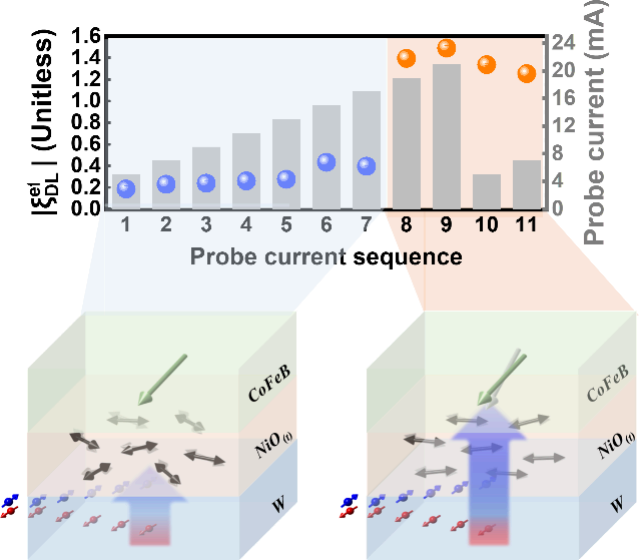

Spin transport and the associated spin torque effects in antiferromagnets (AFMs) are scientifically interesting but have remained elusive due to the varied observations of spin transport in AFMs. This study revisits the role of a global Néel order in nickel oxide (NiO) facilitated through a spin–orbit torque (SOT) and examines the enhanced SOT efficiency in a heavy metal (W)/AFM (NiO)/ferromagnet (FM, CoFeB) trilayer with varying NiO thicknesses ranging from 1 to 5 nm. At the as-grown state, the Néel order of NiO is randomly oriented due to the polycrystalline nature of the film structure, leading to increased spin absorption and blocking spin transport from the adjacent W layer. When the spin current amplitude exceeds a threshold value, SOT enables reorientation of the Néel order in NiO to an equilibrium state, forming a global Néel order aligned with the applied current. This long-range Néel order reduces spin absorption and enhances spin transport through NiO, hence boosting the SOT efficiency in the adjacent CoFeB layer. X-ray magnetic linear dichroism spectroscopy and rewritable Néel order reorientation experiments in a device with orthogonal geometry confirmed the strong correlation between the global Néel order facilitation and the boosted SOT efficiency, which is enhanced larger than 4-fold for both damping- and field-like torques in the trilayer with 5 nm NiO. This study not only reveals the strong correlation between globally facilitated Néel order and spin transport in NiO but also offers a promising manner to promote AFM-based SOT devices toward energy-efficient computing technology.

## 1 Introduction

Antiferromagnets (AFMs) have currently emerged as a novel and significant branch of spintronics, prominent for their non-trivial properties resulting from the strong correlation between the specific AFM order and spintronic dynamics, such as the observation on the anomalous Hall effect and the magnetic spin Hall effect.^1−6^ Traditional research has predominantly focused on manipulating the AFM order to generate a spin current and studying the associated spin torque effects, typically achieved using external fields at accessible levels.^7−10^ As a benefit from these distinctive spintronic dynamics, integrating AFMs with modern spin–orbit torque (SOT) technology based on a heavy metal/ferromagnet (FM) bilayer has been shown to open several possibilities with respect to the ultrafast switching dynamics,^11−13^ enhanced thermal stability,^14−16^ peculiar SOT ratchets,^17−19^ and potential memristivity for neuromorphic applications.^5,20−23^ Moreover, inserting an AFM insulator between the heavy-metal and FM layers has demonstrated remarkable spin transparency and even spin amplification in several model systems,^24−26^ which has been considered associated with the suppressed spin back flow at the AFM interface and spin memory loss in the AFM bulk.^27^ It has been observed that an AFM insulator like NiO, with a moderate thickness of approximately 1 nm, enables enhancing the spin transmission significantly;^28−30^ however, further increasing the AFM thickness tends to suppress spin transmission.^24,25,28−30^ The prevailing explanation attributes spin transport in AFM insulators to the magnon dynamics, driven by spin fluctuations near the Néel temperature of the AFM.^24,28,29,31^ Thus, increasing the AFM thickness to restore AFM robustness appears to suppress spin fluctuations and consequentially reduce spin transmission. However, notable spin transmission has also been reported in a Bi_2_Se_3_/NiO/NiFe trilayer system, even with ultrathick NiO layers around 25 nm, and other related literature.^24−26^ This discrepancy highlights the ongoing debate regarding the optimal NiO thickness for effective magnon transport, which should be comprehensively understood to promote related technologies using AFMs.

This study attempts to revisit the interplay between spin transport and the Néel ordering of AFM and the associated SOT effect in a W/NiO/CoFeB trilayer. A critical aspect under investigation in the recent focus is how the AFM Néel order interacts with SOT and, reciprocally, how the SOT can be utilized to establish a global Néel order in AFMs, thereby enhancing the SOT efficiency.^32^ Our findings show that both damping- and field-like torque efficiencies in the trilayer device with the established global Néel order are substantially amplified in comparison to the device without such ordering. With deviation from the current approaches of enhancing the SOT efficiency, such as strain engineering,^33^ interfacial modification by spacer layers,^34^ collaborative orbital and spin Hall effects,^35^ electrically assisted piezoelectric effect,^36^ and optical modulation,^37^ this study proposes an intrinsic switch to tailor spin conductivity in AFM oxide through the control of its magnetic order, which unveils the magnetic facet of tailoring SOT efficiency in the heterostructure. This research not only addresses the ongoing controversy regarding spin transparency in NiO via the dimension of tailoring the global AFM order but also opens an avenue toward developing low-power SOT devices in the future.

## 2 Results

**Figure 1:**
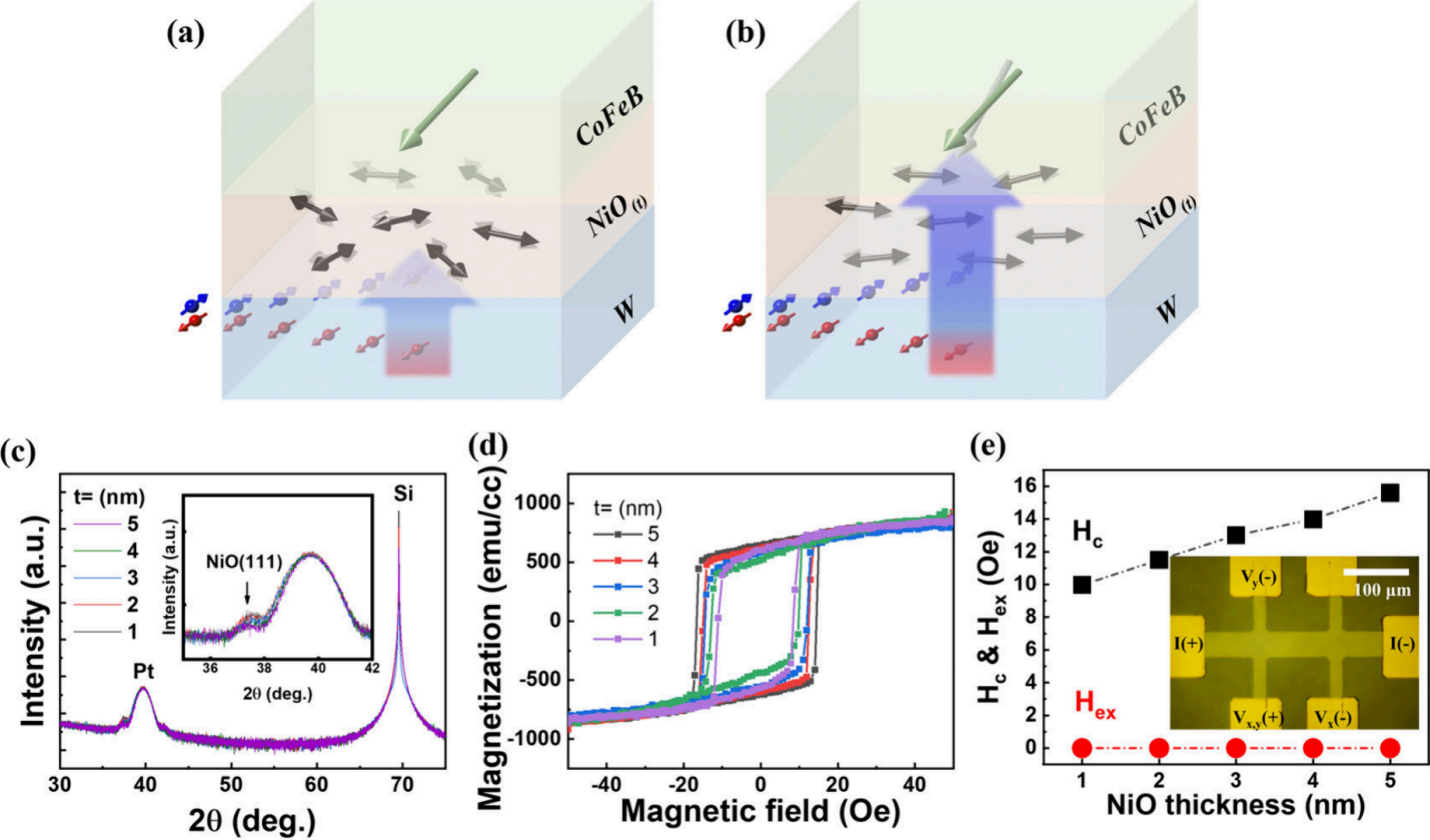
(a) Schematic diagram to show the randomly orientated Néel order and the spin blocking effect reflected on the local moment perturbation in the NiO layer due to the strong spin absorption. (b) Schematic diagram to show the facilitated global Néel order through increasing the SOT strength with increasing the applied current amplitude. Once the global Néel order in NiO is facilitated, the reduced spin absorption should promote the SOT efficiency on the amplified interaction with the magnetization in the CoFeB layer. (c) Synchrotron-based HRXRD patterns for verifying (111)-textured NiO with varying NiO thicknesses (*t*). (Inset) Highlighted HRXRD peaks at NiO(111). (d) Magnetic hysteresis curves of the Pt/NiO(*t*)/CoFeB trilayer with *t* ranging from 1 to 5 nm. (e) Plots of *H*_c_ and *H*_ex_ versus NiO thickness obtained and converted from panel d. The inset shows the device geometry to measure the magnetoresistance and Hall effect along the longitudinal channel and transverse channel, respectively.

Panels a and b of Figure 1 schematically depict the basic concept of studying spin transport and the associated SOT effects in the heavy metal (W)/AFM (NiO)/FM (CoFeB) trilayer. As shown in Figure 1a, the as-deposited trilayer exhibits randomly oriented Néel orders in NiO with easy-plane anisotropy due to its polycrystalline nature fabricated using a sputtering technique, where the local Néel orders are determined via the local AFM anisotropy. When a moderate spin current is generated from the bottom W layer, the subsequent spin current transporting into NiO would be absorbed by the AFM moments and transfer the spin angular momenta to NiO’s moments, hence leading to the moment perturbation in NiO. With the increase of the spin current amplitude via the charge current applied into the W layer, the SOT drives the Néel order to reorient to the equilibrium state with a global Néel order aligned collinearly with the current based on the SOT-driven spin–flop-like transition of NiO (see Supporting Information 1).^38−40^ Consequently, the facilitated global Néel order in NiO reduces the spin passivation and, subsequently, promotes spin transparency through the NiO layer without sacrificing the SOT amplitude. This may involve a similar mechanism, where the weighting between spin absorption and transmission in the NiO layer can be adjusted through the switching from randomly oriented to globally aligned Néel order. Therefore, the SOT efficiency is anticipated to be sustainable or even boosted in comparison to the NiO-free system once the global Néel order is facilitated by SOT, which is technically achievable by increasing the current amplitude in the bottom W layer.

To understand the correlation between the SOT-facilitated global Néel order in the NiO layer with varying magnetic robustness and the associated SOT effects on the top FM layer, W/NiO(*t*)/CoFeB trilayers with varying NiO thicknesses from 1 to 5 nm were fabricated. On the basis of the results of synchrotron-based high-resolution X-ray diffraction (HRXRD), as shown in Figure 1c, sputtered NiO appears to be (111)-textured along the perpendicular direction, which is consistent with observations in similar systems grown by sputtering,^41,42^ enabling an easy-plane anisotropy in NiO for studying the Néel order reorientation in the subsequent section. Figure 1d exhibits the hysteresis curves of the W/NiO(*t*)/CoFeB trilayer with varying *t*. The saturation magnetization of the trilayer did not change significantly in response to changes in the NiO thickness. Figure 1e plots the coercivity (*H*_c_) and exchange bias (*H*_ex_) versus NiO thickness. It shows that *H*_c_ increased monotonically with increasing NiO thickness, and no distinguishable *H*_ex_ was observed. The results suggested that the AFM ordering in NiO at 5 nm was not robust enough to immobilize the interfacial spins; hence, no *H*_ex_ was observed. This minimizes the effects of *H*_ex_ on SOT efficiency,^24,43^ allowing the focus to be solely on the effects of AFM robustness and ordering in NiO for the following SOT investigation.

**Figure 2:**
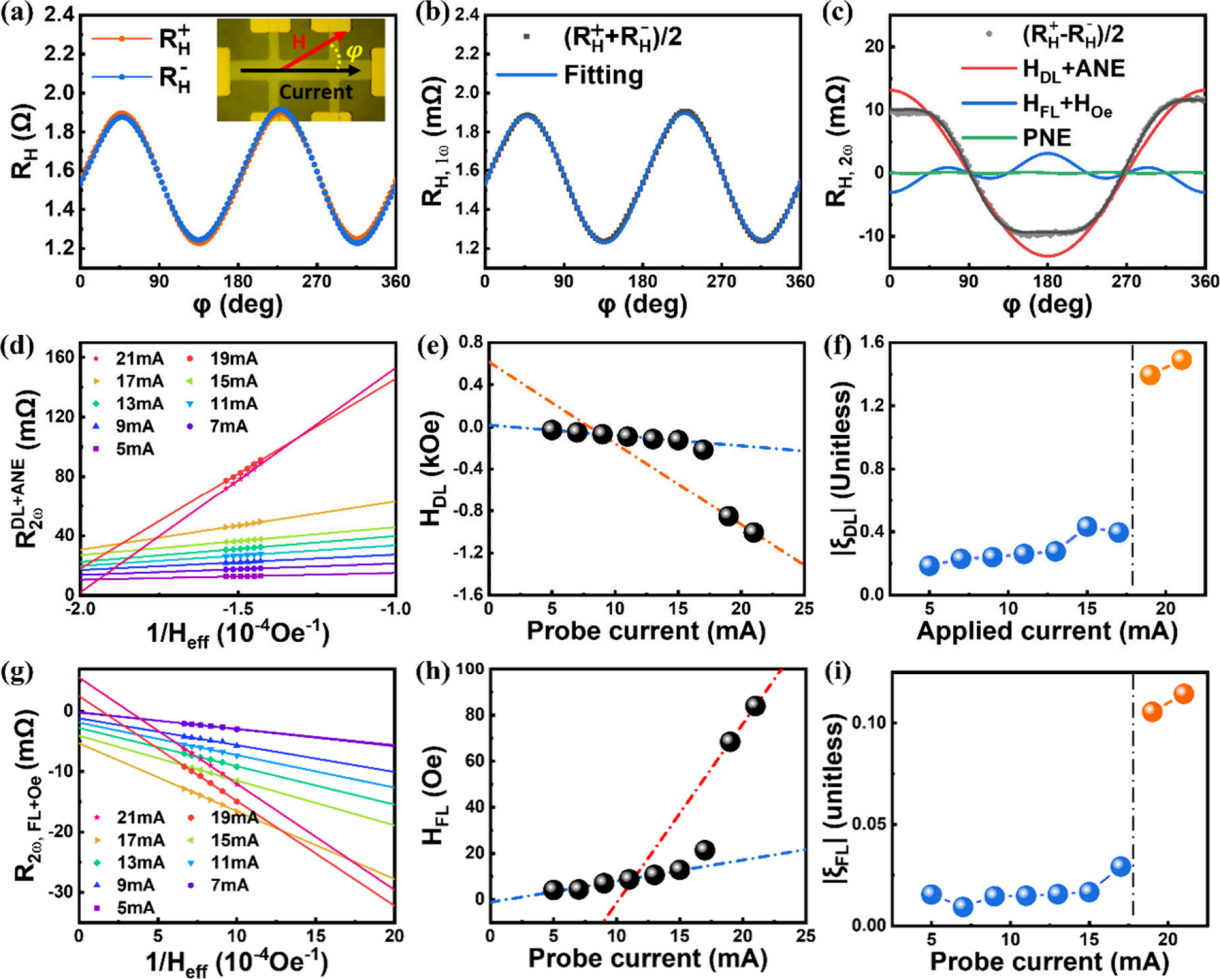
(a) Angle (φ)-dependent Hall resistance of the trilayer device probed by ±5 mA, denoted as *R*_H_^+^ and *R*_H_^–^. (Inset) Experimental geometry of the angle-dependent Hall resistance measurement with applying a field (*H*) rotating anticlockwise with an angle (φ) relative to the applied probe current at +5 mA. (b) First harmonic Hall signal obtained from (*R*_H_^+^ + *R*_H_^–^)/2 with the φ dependency. (c) Second harmonic Hall signal obtained from (*R*_H_^+^ – *R*_H_^–^)/2 with the φ dependency, comprising three components, including the damping-like effective field (*H*_DL_) and anomalous Nersnt effect (ANE), the field-like effective field (*H*_FL_) and current-induced field (*H*_Oe_), and the planar Nersnt effect (PNE). (d) Plots and fitting for *H*_DL_ and ANE extracted from the second harmonic Hall with various probe amplitudes. (e) Extracted *H*_DL_ induced as a function of the probe current amplitude. (f) Converted damping-like torque efficiency (ξ_DL_) as a function of the probe current amplitude. (g) Plots and fitting for *H*_FL_ and *H*_Oe_ extracted from the second harmonic Hall with various probe amplitudes. (h) Extracted *H*_FL_ induced as a function of the probe current amplitude. (i) Converted damping-like torque efficiency (ξ_FL_) as a function of the probe current amplitude.

Figure 2a shows the angle (φ)-dependent Hall signals acquired with ±5 mA, denoted as *R*_H_^+^ and *R*_H_^–^, taken from the device with NiO(5). The inset in Figure 2a depicts the experimental geometry for the φ-dependent harmonic Hall measurements with an applied external field (*H*), ranged from 1000 to 1500 Oe, with an angle (φ) relative to the applied current of +5 mA in the device plane. SOT arising from the bottom W layer would slightly tilt the magnetic moment out of plane and toward transverse directions of the device, driven by damping- and field-like torques, respectively, thus resulting in an additional Hall signal to make the planar Hall curves shifted. Panels b and c of Figure 2 display the first and second harmonic Hall signals, defined as (*R*_H_^+^ + *R*_H_^–^)/2 and (*R*_H_^+^–*R*_H_^–^)/2, respectively. The first harmonic signal represents the spin-independent term used as a reference for subsequent SOT efficiency analyses. The second harmonic signal represents the spin-dependent characterization, used to extract the SOT effect by decomposing it into several components, including the combined damping-like effective field (*H*_DL_) and the anomalous Nernst effect (ANE, red line), the entangled field-like effective field (*H*_FL_) and the current-induced Oersted field (*H*_Oe_) (blue line), and the planar Nernst effect (PNE, green line).^44−47^ After the second harmonic signals were fit with an overall deviation smaller than 4% (see Supporting Information 2), both *H*_DL_ and *H*_FL_ and the associated torque efficiencies could be extracted.

Figure 2d plots the extracted second harmonic signal for *H*_DL_ and ANE obtained with various probe current amplitudes. The slopes of the plots represent *H*_DL_ after subtracting the contribution of ANE as the intercept in the *R*_2ω_^DL,ANE^ versus (*H*_ext_ – *H*_k_^eff^)^−1^ correlation, in which *H*_ext_ ranged from 1000 to 1500 Oe and *H*_k_^eff^ was approximately 7340 Oe, obtaining an anomalous Hall effect measurement with a perpendicular field (*H*_*Z*_) (see Supporting Information 3). Panels e and f of Figure 2 show the evolution of *H*_DL_ and the resulting efficiency (ξ_DL_) with an increasing probe current amplitude. Both *H*_DL_ and ξ_DL_ were observed to increase gradually in response to the moderate probe current amplitudes (5–17 mA) and then increase dramatically when the probe current reached 19 mA as a threshold. This sharp increase in SOT efficiency is also supported by the magnetoresistance (MR)-based loop-shift method in y-type geometry (see Supporting Information 4), which is attributed to the Néel order facilitation in NiO examined by X-ray magnetic linear dichroism spectroscopy. It should be noticed that the overall ξ_DL_ appears to follow a specific current dependence with a positive correlation. This property can be attributed to the distributed Néel orders, which naturally exhibit varying local anisotropy strengths. As a result, the primary population of Néel order would be responsible for the threshold current, but the subordinate Néel orders might respond differently to the applied currents, hence leading to the observed slight increase in ξ_DL_ with increasing the current amplitude before and after reaching the threshold. In addition, the effects of facilitating a global Néel order in NiO on the boosted SOT efficiency were also reflected in the field-like torque efficiency (ξ_FL_), after subtracting the *H*_Oe_ component in the second harmonic signal, as shown in panels g–i of Figure 2. The similar evolution in *H*_DL_ (ξ_DL_) and *H*_FL_ (ξ_FL_) versus probe current amplitude suggests a common origin of SOT from the bottom W layer and its modification through NiO. This phenomenon, with respect to the boosted SOT efficiency, was also observed in the Pt/NiO(*t*)/CoFeB trilayer (see Supporting Information 5), where the bottom SOT source was replaced by Pt. The result suggests that the enhanced SOT efficiency driven by the facilitated Néel order should be a universal phenomenon. It should be noticed that, although the external field applied for the harmonic Hall measurement might alter the Néel order orientation in the NiO layer during the angle-dependent scan, the broaden distribution of Néel order strengths might still make partial local AFM domains unaltered to preserve the spin configuration as Figure 1b;^48,49^ hence, the boosted SOT efficiency was observed. In addition, the SOT efficiency examined using the MR-based loop-shift method in y-type geometry (Supporting Information 4) shows a similar trend before and after the Néel order setting, with a clear dependence upon the NiO thickness. In this method, the field reversal was confined to the *y* direction of the device, which consistently favored the alignment of the Néel order along the *x* direction via the spin–flop dynamics. The similar trend in SOT efficiency observed between the harmonic Hall measurement and the loop-shift method suggests a common origin, indicating that the facilitation of the Néel order may dominate the enhancement on the SOT efficiency.

**Figure 3:**
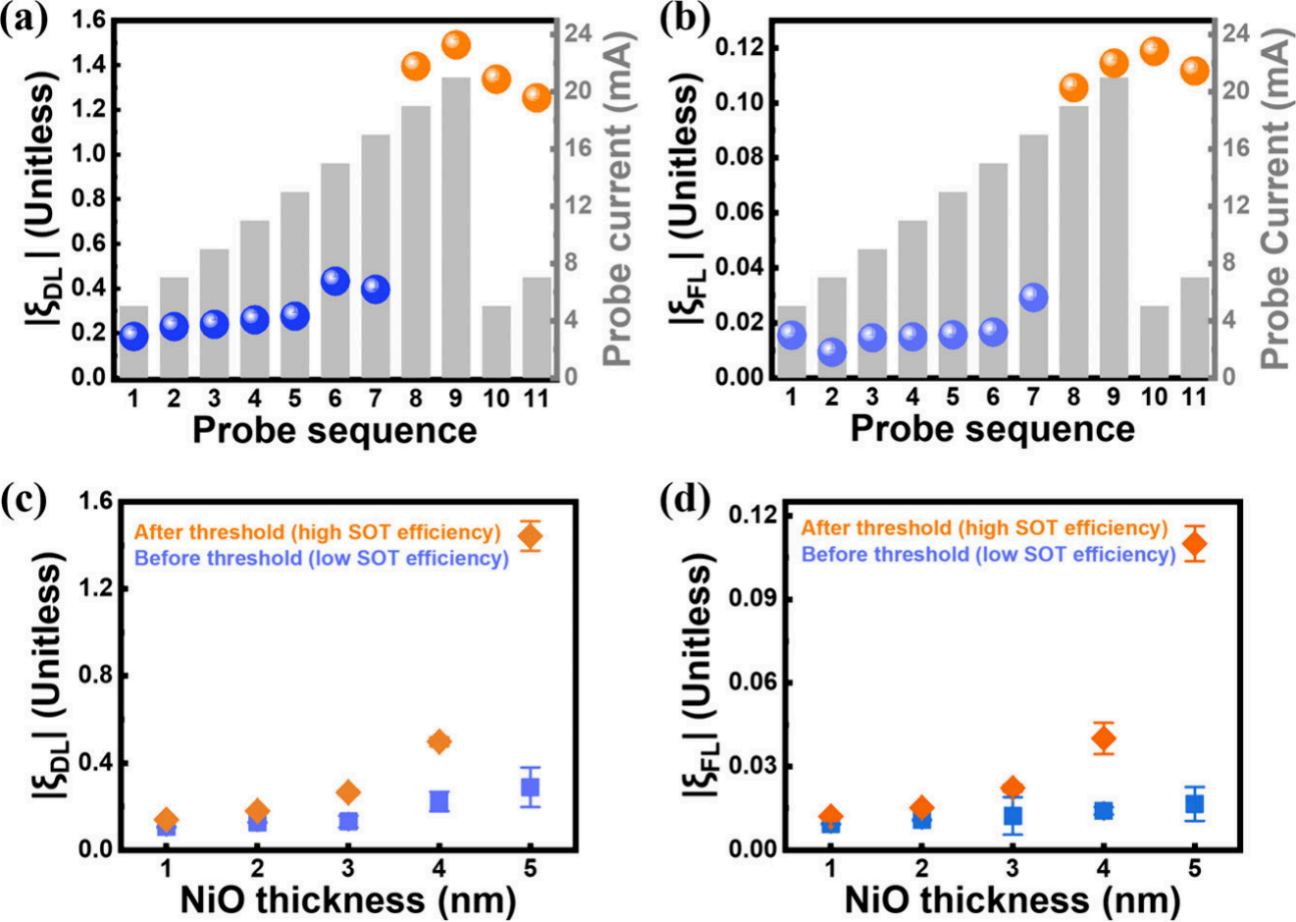
(a) Absolute value of ξ_DL_ and (b) ξ_FL_ (colored balls, left axis) of the trilayer with NiO(5) obtained by varying the probe current amplitude (charts, right axis). After a threshold current at ∼19 mA, both ξ_DL_ and ξ_FL_ appear to be at a sustainable level even though the probe current is reduced. (c) Absolute value of ξ_DL_ and (d) ξ_FL_ as a function of the NiO thickness probed before and after treating a threshold current of ∼19 mA, taken by averaging the SOT efficiencies at the low (blue dots) and high (orange dots) SOT efficiency state in panels a and b with error bars covering the variation.

Although the results in Figure 2 have shown that the SOT efficiencies (both ξ_FL_ and ξ_DL_) could be significantly boosted, a potential issue related to the thermal effect driven by the larger probe current may influence the fitting result. To address this issue, panels a and b of Figure 3 show the plots for ξ_DL_ and ξ_FL_ of the trilayer with NiO(5) obtained by gradually increasing the probe current amplitude to 21 mA and subsequently reducing it to 5 mA, as demonstrated by the gray chart in each panel. Surprisingly, both ξ_DL_ and ξ_FL_ appear to remain boosted after removal of the high probe current (21 mA). Two important features can be revealed from the results in panels a and b of Figure 3: (1) The SOT efficiency had been permanently changed once a large probe current was applied to the trilayer system. (2) The Joule heating effect may not play a major role responsible for the boosted SOT effect, because the effect was still observed when using a smaller probe current to measure the SOT efficiency later.

In addition to the boosted SOT efficiency along with considerable non-volatility, the systematic investigations of the boosted SOT efficiency on ξ_DL_ and ξ_FL_ as a function of the NiO thickness are exhibited in panels c and d of Figure 3, respectively. Both ξ_DL_ and ξ_FL_ increase significantly after treating with a pulse current of 19 mA for all of the investigated devices, particularly for the trilayer with NiO(5), which shows approximately 400 and 563.7% enhancement for ξ_DL_ and ξ_FL_, respectively. ξ_DL_ (∼0.13) and ξ_FL_ (∼0.012) of the trilayer with NiO(1) were even larger than those of the NiO-free device with ξ_DL_ (∼0.05) and ξ_FL_ (smaller than the resolution of facility of <1 × 10^–3^). Furthermore, ξ_DL_ and ξ_FL_ versus NiO thickness obtained after treatment with a pulse current of 19 mA increase parabolically up to NiO(5). This suggests that the increased AFM periodicity with well-facilitated Néel ordering should be able to promote spin transport in NiO, thus resulting in boosted SOT efficiencies.

**Figure 4:**
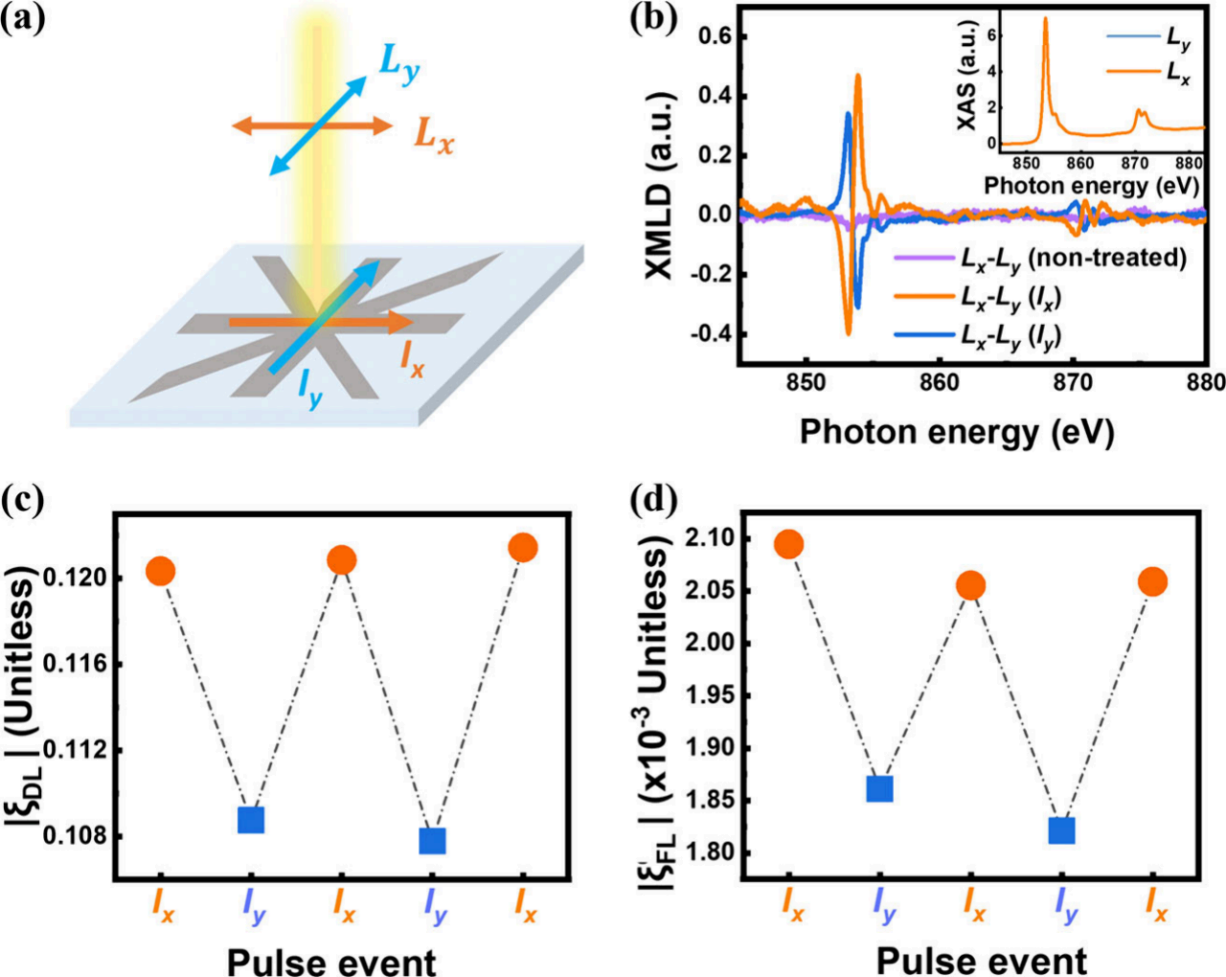
(a) Experimental geometry for the measurement of XMLD and the rewritable SOT efficiency characterization on an eight-terminal device with NiO(3). *L*_*x*_ and *L*_*y*_ stand for the X-ray incidences with a linear polarization along *x* and *y* directions, respectively, which also corresponds to *x* and *y* directions of the device. (b) XMLD spectra of the NiO(3) device acquired from the difference between the absorption spectra using *L*_*x*_ and *L*_*y*_ without treating the current (purple) and after treating a threshold pulse current along *x* (orange) and *y* (blue) directions of the device, denoted as non-treated, *I*_*x*_, and *I*_*y*_, respectively. (c) Absolute value of ξ_DL_ and (d) ξ_FL_ acquired after treating *I*_*x*_ and *I*_*y*_ alternatively, which show reversible changes between two distinguishable states.

To verify the changes in the Néel order in NiO after treatment with the probe current and the associated effects on the SOT efficiency, X-ray magnetic linear dichroism (XMLD) spectroscopy was employed to observe the Néel order transition in NiO. Figure 4a presents the schematics of the XMLD measurement. An eight-terminal-patterned device was utilized to reorient the Néel order of NiO along two orthogonal axes using a purge pulse current (*I*_*x*_ and *I*_*y*_) above the threshold to observe the boosted SOT efficiency. The XMLD spectra at the Ni *L*_2_/*L*_3_ edges were acquired from the difference between two X-ray absorption spectra taken by X-ray with linear polarization along the *x* and *y* directions, denoted as *L*_*x*_ and *L*_*y*_ in Figure 4a. Figure 4b shows the results of XMLD (*L*_*x*_ – *L*_*y*_) after treating the device with *I*_*x*_ and *I*_*y*_. The trilayer device with NiO(3) was employed for the XMLD measurement, enabling probing the entire NiO layer owing to the surface-sensitive nature of XMLD of ∼5 nm using a total electron yield mode. As a result, the completely symmetric XMLD spectrum can be reversed by alternatively applying *I*_*x*_ and *I*_*y*_, indicating the reversible Néel order reorientation between the *x* and *y* axes. On the basis of related literature utilizing XMLD to define the Néel order direction, it appears that the Néel order of NiO aligns with the axis collinear to the applied current, indicating that spin–flop dynamics may dominate the Néel order reorientation during the SOT application.^38−40^ The observed result is also opposite the literature regarding the Néel order reorientation transverse to the current (*y* direction) in NiO driven by the current-induced thermal effect,^50−54^ which consequently eliminates heating-associated effects in our study. Notably, the XMLD measurement was not performed *in situ* right after treating the probe current. Therefore, the observed Néel order reorientation on XMLD is permanent, which may be responsible for the non-volatile SOT efficiency enhancement shown in panels a and b of Figure 3.

To further investigate the effects of Néel order reorientation in NiO on the SOT efficiency along the two orthogonal axes, panels c and d of Figure 4 plot ξ_DL_ and ξ_FL_ acquired along the *x* direction after alternately applying *I*_*x*_ and *I*_*y*_. It shows that both ξ_DL_ and ξ_FL_ vary between two distinguishable levels upon treating *I*_*x*_ and *I*_*y*_. Driving the Néel order along the longitudinal (*x*) direction results in high SOT efficiency, whereas the Néel order along the transverse (*y*) direction results in low SOT efficiency, effectively acting as a SOT switch to adjust spin conductivity in NiO. Note that the SOT efficiencies in panels c and d of Figure 4 were obtained from the trilayer device with NiO(3) for matching the XMLD results in Figure 4b. The results align well with the conceptual scheme in Figure 1b. Therefore, once the global Néel order of the AFM is established and well-defined, the spin conductivity can be significantly boosted along with the amplified SOT efficiency. These findings are not only scientifically significant but also practically useful for AFM-based SOT devices aimed at low-power consumption.

## 3 Discussion

Thus far, it has been demonstrated that a well-facilitated Néel order in NiO enables the adjustment of its spin conductivity. However, the two remaining issues still require further understanding. First, the two distinguishable levels in panels c and d of Figure 4 did not exhibit an ideal SOT switch with on–off characteristics. This suggests that the established Néel order in the orthogonal geometry still allows for partial spin transport instead of full suppression. However, the detectable SOT efficiency in the orthogonal geometry, as indicated by the blue dots in panels c and d of Figure 4, may result from the geometrical effect, such as the issue of current distribution in the eight-terminal device (see Supporting Information 6). This means that the non-switched part at the vicinity of the Hall terminals may still contribute to the SOT effect; therefore, the efficiency obtained after the *y*-directional rewriting is still quantifiable, as demonstrated in panels c and d of Figure 4. Additionally, the trilayer with NiO(3) was employed for the orthogonal rewriting examination due to its suitability for XMLD measurements across the full stack of sub./W(3)/NiO(*t*)/CoFeB(2)/Pt(1). Given the surface-sensitive nature of XMLD, which probes a depth of approximately 5 nm, the trilayer with NiO(3) allowed the examination of the entire NiO layer in the stack. Therefore, the SOT efficiencies for comparison should be 0.26 ± 0.001 and 0.015 ± 0.009 for ξ_DL_ and ξ_FL_ in panels c and d of Figure 3, respectively, which are comparable to the values around ∼0.12 and ∼0.0021 for ξ_DL_ and ξ_FL_ in panels c and d of Figure 4. Thus, modification of the device geometry should be considered in the next phase to address this issue more comprehensively. Moreover, it has been shown that the trilayer device with 5 nm NiO yielded the largest SOT efficiency after the global Néel order facilitation. In comparison to the previous literature, the prevailing elaboration on the spin transport in AFM oxides was correlated with the magnon or spin fluctuation near the Néel temperature.^24,27,28,30^ The insertion of AFM oxides appears to be capable of suppressing the spin back flow at the AFM interface and spin memory loss in the AFM bulk.^27^ Furthermore, the specific magnetization dynamics of AFM may also enable the SOT amplification, in which the spin accumulation at the AFM interface may generate a strong spin torque due to ultrafast dynamics on the ∼terahertz (THz) scale; that is, the torque defined as d*m*/d*t* would be large if d*t* is relatively short, as in AFMs. Consequently, the precession of the two sublattice spins in the AFM could propagate a large spin torque through the entire AFM, hence amplifying the SOT effect at the FM interface. In this work, it demonstrated that the setting of the global Néel order appeared to be more profound to allow spin transport because the Néel temperature of 5 nm NiO should be higher than room temperature, at which the NiO moments should be more robust against fluctuation. Therefore, our observation should suggest that the precise control over Néel order in AFM oxides should be the most dominant issue for the applications of advanced spintronic devices based on AFMs.

## 4 Summary

This study addresses a previously unresolved issue in the literature regarding spin transport and the associated SOT effect in a W/NiO/CoFeB trilayer system. It has been shown that the divergent observations on the spin transport length in NiO may originate from varying degrees of Néel order facilitation and its orientation relative to the polarity of the spin current. When the Néel order of NiO is well-facilitated using SOT, in which the Néel order is aligned orthogonal to the spin polarization, it significantly boosts the SOT efficiency. This is due to the equilibrium Néel order state driven by SOT, which gives rise to suppressed spin absorption during spin transport through NiO. The facilitated Néel order promotes the SOT efficiency, functioning as a SOT booster, a phenomenon observed universally in all investigated devices with varying NiO thicknesses from 1 to 5 nm and with different spin current layers as Pt. The enhanced SOT efficiency in NiO(5) was found to increase approximately 4-fold after such Néel order facilitation. Therefore, utilizing AFM NiO with appropriate Néel order manipulation functions as a SOT booster, enhancing SOT efficiency, and potentially driving NiO-based SOT devices toward energy-efficient device technologies.

## 5 Materials and Methods

### 5.1 Materials and Device Fabrications

Film stacks of Si/SiO_2_/W(3)/NiO(*t*)/CoFeB(2)/Pt(1) with varying thicknesses of NiO, *t* = 1, 2, 3, 4, and 5 nm, were deposited using a magnetron sputtering technique in a chamber of 3 × 10^–8^ Torr basal pressure, where the numbers in parentheses for each layer stand for the thickness in nanometers. W, Pt, and CoFeB were grown by direct current (DC) sputtering with a power of 30 W (for W and Pt) and 50 W (for CoFeB) under an Ar pressure under 3 mTorr. NiO was grown by radio-frequency (RF) sputtering with a power of 150 W. During the deposition process, the substrate holder was rotated at 10 rpm for ensuring the film homogeneity. After deposition, all of the sheet film samples were magnetically characterized at 300 K using a vibrating sample magnetometer in a physical property measurement system (Quantum Design) and then patterned into Hall bar devices of 160 μm in length and 20 μm in width using an Ar ion-beam etching facility. Before Ti(5)/Au(105) electrodes were deposited using an E-gun evaporation, the terminals of the device for electrode contact were pre-sputtered to remove the stack above the W layer to allow the direct contact between W and Ti(5)/Au(105) electrodes to avoid current shunting during the electrical measurements. The prepared Hall bar device geometry for the SOT-associated investigations was shown as the inset in Figure 1d. The process of the eight-terminal device was similar to that of the Hall bar, but in addition to the orthogonal cross structure with 20 μm in width for applying currents, the device geometry required two additional diagonal channels with 5 μm in width for detecting the Hall signal.

### 5.2 Angle-Dependent Harmonic Hall Measurements

Harmonic Hall measurements were performed using a GMW Associates 5204 vector projected field electromagnet platform. The measurement began with applying a direct current with varying amplitudes along the *x* direction of the device using a Keithley 2400 sourcemeter together with applying a magnetic field (*H*) with an angle (φ) rotating anticlockwise in the device plane and relative to the current direction. The resulting Hall voltage changes during the *H* rotation was collected using a Keithley multimeter. The probe current amplitude was varied from ±5 to ±21 mA and repeated in the range to observe the non-volatile effects after treating the probe currents with different amplitudes. The *R*^2^ analyses on the fitting of gathering *H*_DL_ and *H*_FL_ from the harmonic Hall measurements were examined to demonstrate the reliability of data analysis.

### 5.3 X-ray Magnetic Linear Dichroism

The XMLD spectra acquired at Ni *L*_2,3_ edges were performed at 45A1, Taiwan Photon Source (TPS), National Synchrotron Radiation Research Center (NSRRC), Taiwan. The X-ray absorption spectroscopy (XAS) spectra were collected in total-electron yield (TEY) mode by normally shining the X-ray with a linear polarization parallel (*L*_*x*_) and orthogonal (*L*_*y*_) to the device channel of the applied current (*I*_*x*_). The XMLD spectra were obtained from the difference between the XAS done by *L*_*x*_ and *L*_*y*_, *L*_*x*_ – *L*_*y*_, after treating each pulse current *I*_*x*_ and *I*_*y*_ alternatively. The spot size of the X-ray was focused to 3 × 3 μm [full width at half maximum (FWHM)], enabling a precise probe on the intersection region of the device to see the spectroscopic changes after treating *I*_*x*_ and *I*_*y*_.

## Supporting Information Available

|SOT-driven Néel order reorientation (Supporting Information 1), *R*^2^ analysis for the fitting accuracy of harmonic Hall signals (Supporting Information 2), subtracting the ANE signal from second harmonic Hall measurements (Supporting Information 3), SOT efficiency characterized using a MR-based loop-shift method in y-type geometry (Supporting Information 4), SOT efficiency characterizations for the Pt/NiO(*t*)/CoFeB trilayer (Supporting Information 5), and geometrical effect of the eight-terminal device on the orthogonal rewriting examination (Supporting Information 6) (PDF)
