# Supplementary material for: Spin–Orbit Torque Booster in an Antiferromagnet via Facilitating a Global Antiferromagnetic Order: A Route toward an Energy-Efficient Memory
Source: ACS Appl Mater Interfaces. 2024 Nov 14;16(47):65037–45. doi: 10.1021/acsami.4c15453 (PMC11615842; doi:10.1021/acsami.4c15453)
Supplement: Supplementary file 1 — am4c15453_si_001.pdf [file am4c15453_si_001.pdf]

(Supporting Information)

A Spin-Orbit Torque Booster in Antiferromagnet via Facilitating a Global Antiferromagnetic Order: A Route Toward an Energy-efficient Memory

Hao-Kai Chang[1], Kuan-Yu Chi[2], Yu-Lon Lin[1], Yu-Hsien Lai[1], Yen-Lin Huang[1,3], Chi-Feng Pai[2]*, Chao-Yao Yang[1,3]*

[1]*Department of Materials Science and Engineering National Yang Ming Chiao Tung University Hsinchu 300093, Taiwan.*

[2]*Department of Materials Science and Engineering, Center of Atomic Initiative for New Materials, and Center for Quantum Science and Engineering, National Taiwan University, Taipei 10617, Taiwan.*

[3]*Center for Emergent Functional Matter Science, National Yang Ming Chiao Tung University, Hsinchu 300093, Taiwan*

To whom correspondence should be addressed: cfpai@ntu.edu.tw; cyyang8611@nycu.edu.tw

Supporting information 1 | SOT-driven Néel order re-orientation

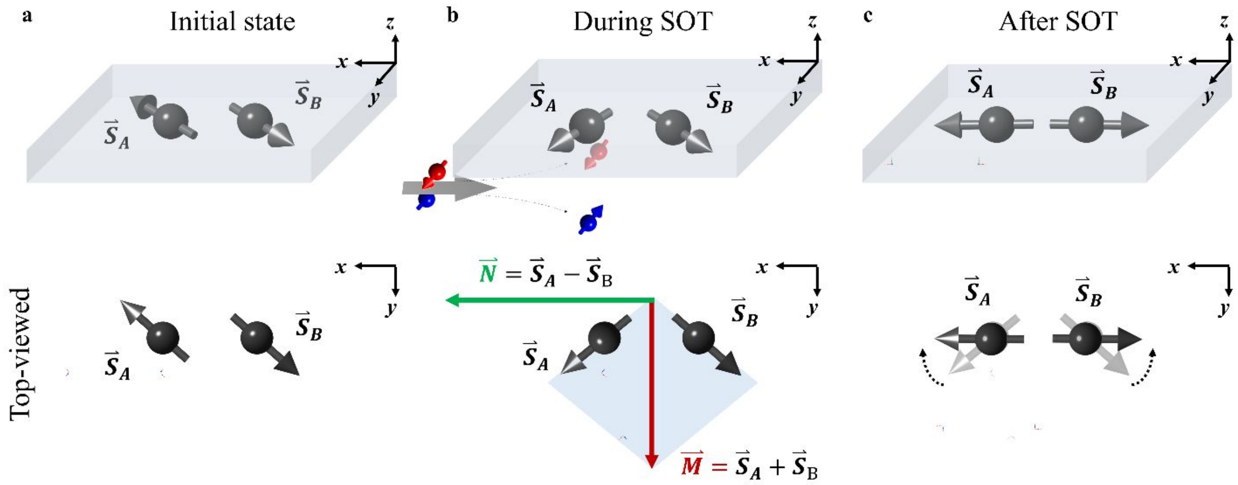

Figure S1 Schematic diagram to exhibit the Néel order transition at **a**, the initial state, **b**, the state during the SOT applied, and **c**, the state after SOT. Figures at the bottom of each panel exhibit the top-viewed image of the two-spin configurations corresponding to the different SOT stages.

In this study, the Néel orders of NiO should randomly orientate in the film plane at the as-deposited state because of the absent anisotropy in the poly-crystalline film structure. At this stage, the local Néel order is allowed and determined by the local anisotropy in a NiO grain. **Figure S1a** shows a pair of AFM sub-lattice spins, denoted as $\vec{S}_A$ and $\vec{S}_B$, in the film plane, which are aligned deviating from the x- and y-axis at the initial state. Note, the pair of AFM sub-lattice spins could be pointed randomly in the plane, which would not reverse the conclusion on the global Néel order facilitation by SOT in the following discussion. Upon applying the current for the SOT from the bottom, the field-like torque would drive both $\vec{S}_A$ and $\vec{S}_B$ toward the transverse direction in the form of a spin-flop-like transition during the SOT. This spin-flop-like dynamics is to reduce the overall Zeeman energy once the induced ferromagnetic moment, defined by $\vec{M} = \vec{S}_A + \vec{S}_B$, is rotated by the field-like torque along the y-direction[1-5]. Consequently, the Néel order defined by $\vec{N} = \vec{S}_A - \vec{S}_B$ would be pushed toward the x-direction based on the orthogonal geometry with $\vec{M}$, as shown at the bottom of **Figure S1b**. After the SOT treatment, the two spins would return to x-axis as shown in **Figure S1c**, giving rise to the observation on the global Néel order facilitated along the x-direction upon collecting all the local Néel orders distributed in the NiO layer. Note: The damping-like effective field would drive both $\vec{S}_A$ and $\vec{S}_B$ rotate coherently in the x-z plane during the SOT pulse. After SOT, $\vec{S}_A$ and $\vec{S}_B$ would return to the x-y plane due to the easy-plane anisotropy.

Supporting information 2 | R-squared analysis for the fitting accuracy of harmonic Hall signals.

R squared analysis (or coefficient of determination, COD) has been used to determine the fitting accuracy of the harmonic Hall measurements to obtain the damping-like torque efficiency and field-like torque efficiency. The function of the R squared is:

$$R^2 = 1 - \frac{SS_{res}}{SS_{tol}} \quad (1)$$

, where SS_{res} and SS_{tol} are the sum of squares of residuals and the total sum of squares defined as follow:

$$SS_{res} = \sum_i (y_i - f_i)^2 \quad (2)$$

$$SS_{tol} = \sum_i (y_i - \bar{y})^2 \quad (3)$$

, where y_i represents the raw data in the number i, f_i represents the fitted data in the number i, and $\bar{y}$ is the mean of the total raw data.

Take the harmonic Hall measurement of the trilayer with NiO(5) acquired using ±5mA probe current under 1000 Oe magnetic field as an example, the fitted 2nd harmonic resistance is $R_{H,2\omega} = R_{2\omega}^{DLANE}\cos\varphi + R_{2\omega}^{FLOe}\cos\varphi\cos 2\varphi + R_{2\omega}^{PNE}\sin 2\varphi$, where the fitting parameter of $R_{2\omega}^{DL,ANE}$, $R_{2\omega}^{FL,Oe}$, and $R_{2\omega}^{PNE}$ are 0.01314, -0.00308, and -1.00794×10^{-4}, respectively. Therefore, the SS_{res} and SS_{tol} can be determined as shown in the table below:

Angle (φ)	0	3		357	Sum (SS_{res})
y_i	0.00975	0.00982		0.00998	-
f_i	0.01006	0.01004	……	0.01008	-
$y_i - f_i$	-3.14337E-4	-2.2533E-4		-1.03314E-4	-
$(y_i - f_i)^2$	9.88077E-8	5.07738E-8		1.06738E-8	1.15483E-5

Angle (φ)	0	3		357	Sum (SS_{tol})
y_i	0.00975	0.00982		0.00998	-
$\bar{y}$	7.33598E-5	7.33598E-5	……	7.33598E-5	-
$y_i - \bar{y}$	0.00967	0.00974		0.00991	-
$(y_i - \bar{y})^2$	9.35534E-5	9.49342E-5		9.81555E-5	0.00822

After calculating the SS_{res} and SS_{tol}, R squared can be determined:

$$R^2 = 1 - \frac{SS_{res}}{SS_{tol}} = 1 - \frac{1.15483\text{E} - 5}{0.00822} = 0.99863$$

After performing R squared analysis to other 2nd harmonic Hall resistances, the damping-like torque efficiency and field-like torque efficiency could be obtained via the well-fitted data with accuracy all higher than 96%, as shown in **Figure S2** series. **Figure S2a** to **Figure S2c** show the same data set as shown in the main content of the manuscript. The rest R squared data acquired by different

probe currents under with various in-plane magnetic fields are demonstrated in **Figure S2d**. All the R squared data is higher than 98.8%, revealing the well-fitted of the 2nd harmonic results including the data points acquired after applying the threshold probe current of 19 mA as highlighted by the open triangles. Moreover, the linear fitting for the plots of $R_{2\omega}^{DLANE}$ - H_{eff} and $R_{2\omega}^{FLOe}$ - H_{eff} correlation represent damping-like effective field and field-like effective field, and the associated torque efficiencies. It shows the R squared of the linear slope in **Figure S2b** and **Figure S2c** is employed to obtain the fitting accuracy of damping-like torque efficiency and field-like torque efficiency, as demonstrated in **Figure S2e** and **Figure S2f**, respectively. Both are higher than 96%, suggesting the reliable fitting results of 2nd harmonic Hall measurements.

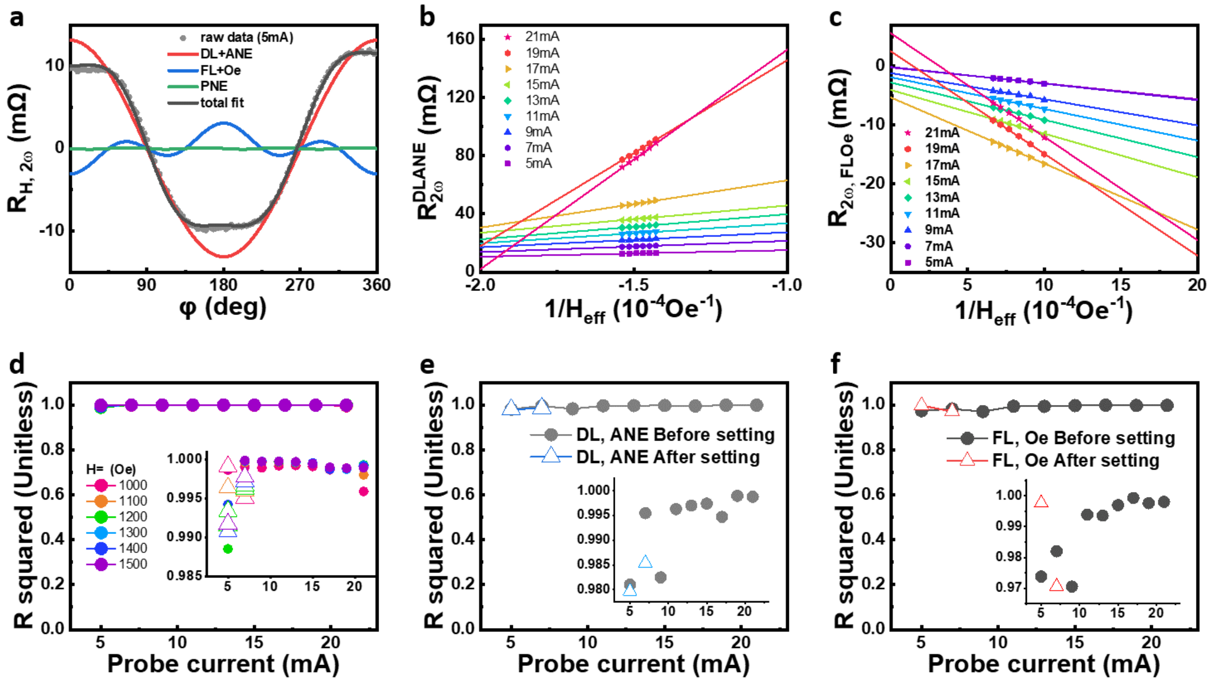

Figure S2 a, 2nd harmonic Hall signal $((R_H^+ - R_H^-)/2)$ of the device with the φ-dependency obtained from the trilayer with NiO(5), comprising three components including damping-like effective field (H_{DL}) and anomalous Nersnt effect (ANE), field-like effective field (H_{FL}) and current-induced field (H_{Oe}), and the planar Nersnt effect (PNE). **b**, Plots and fitting for the H_{DL} and ANE extracted from the 2nd harmonic Hall with various probe amplitudes. **c**, Plots and fitting for the H_{FL} and H_{Oe} extracted from the 2nd harmonic Hall with various probe amplitudes. **d**, R squared analysis of the fitting accuracy of the overall 2nd harmonic Hall signal at various probe current amplitudes. **e**, R squared analysis of the fitting accuracy for the H_{DL} and ANE at various probe current amplitudes. **f**, R squared analysis of the fitting accuracy for the H_{FL} and H_{Oe} at various probe current amplitudes. Insets in **d**, **e**, and **f**, show the enlarged images of the plots in the main panel and the open triangles mark the fitting accuracy after

applying the threshold probe current of 19 mA for examining the non-volatility, in which the fitting accuracies are all higher than 96%.

Supporting information 3 | Subtracting ANE signal from 2nd harmonic Hall measurements

Harmonic Hall measurement is a precise approach to characterize the spin-orbit torque efficiencies in magnetic heterostructures. By applying a probe current with the positive and negative polarity under a certain magnetic field, both 1st harmonic and 2nd harmonic signals can be measured, denoted as equilibrium and non-equilibrium phases of the measurements. The equilibrium phase is dominated by field-dependent transport, reflected on the planar Hall effect (PHE) for an SOT device with in-plane anisotropy. The non-equilibrium phase is dominated by the current-induced effective fields to magnetically bias the magnetic moment deviating from the equilibrium state led by field, which results in additional components in the Hall signal. The equilibrium phase led by a magnetic field in the device plane (x-y plane) is PHE, which can be described as:

$$R_{H,\,1\omega} = R_{PHE}\sin 2\varphi \quad (4)$$

The non-equilibrium phase arises from the combination of damping-like effective field, field-like effective field, and heating-driven effects. Current-induced damping-like effective field can tilt the magnetic moment out-of-plane and insert an additional AHE signal. Besides, current-induced field-like effective field can tilt the magnetic moment toward the transverse direction relative to the current, giving rise to an additional PHE signal. Current-induced joule heating, namely the planar Nernst effect, would slightly affect the fitting result of SOT efficiency. After considering the items aforementioned, the non-equilibrium phase via the 2nd harmonic Hall signal is:

$$R_{H,\,2\omega} = R_{2\omega}^{DL,ANE}\cos\varphi + R_{2\omega}^{FL,Oe}\cos\varphi\cos 2\varphi + R_{2\omega}^{PNE}\sin 2\varphi \quad (5)$$

With the correlation, the 2nd harmonic signal can be decomposed to several terms with φ-dependency. The leading term with $\cos\varphi$ represents the Hall signal led by damping-like torque and ANE, and the middle term with $\cos\varphi\cos 2\varphi$ arises from the field-like torque and Oersted field. The final term with $\sin 2\varphi$ is associated with planar Nernst effect. After the decomposition, Hall signals contributed from H_{DL}, ANE, H_{FL}, H_{Oe}, and PNE can be described by the linear equations as follows:

$$R_{2\omega}^{DL,ANE} = -\,R_{AHE}\cdot H_{DL}\cdot\left(H_{ext} - H_k^{eff}\right)^{-1} + (-\,R_{2\omega}^{ANE}) \quad (6)$$

$$R_{2\omega}^{FL,Oe} = -2R_{PHE}\cdot(H_{FL} + H_{Oe})\cdot(H_{ext})^{-1} \; (7)$$

Therefore, the slope of $R_{2\omega}^{DLANE}$ versus $\left(H_{ext} - H_k^{eff}\right)^{-1}$ correlation enables extracting the H_{DL}. The R_{AHE} can be acquired via the anomalous Hall measurement with a perpendicular field reversal as demonstrated in **Figure S3a**. R_{PHE} can be acquired via the amplitude of φ-dependent harmonic Hall measurement as demonstrated in **Figure S3b**. R_{ANE} could be obtained via the intercept in the $R_{2\omega}^{DLANE}$ versus $(H_{ext} - H_k^{eff})^{-1}$ correlation and the result was demonstrated in **Figure S3c**. On the counterpart, the slope of $R_{2\omega}^{FLOe}$ versus $(H_{ext})^{-1}$ correlation can resolve the effective field containing the H_{FL} and H_{Oe}, in which H_{Oe} can be obtained based on the equation below:

$$H_{Oe} = \frac{j_e\cdot t_{HM}}{2} \quad (8)$$

, where j_e and t_{HM} correspond to current density and the thickness of the heavy metal layer (SOT

source layer). Therefore, the H_{FL} is determined.

Finally, the ξ_{DL} and ξ_{FL} can be calculated by:

$$\xi_{DL/FL} = \frac{2e\mu_0 M_s t_{FM}}{\hbar}\left(\frac{H_{DL/FL}}{j_e}\right) \quad (9)$$

, where e, μ_0, M_s, t_{FM}, $\hbar$, j_e, $H_{DL/FL}$, and $\xi_{DL/FL}$ are quantity of electron charge, vacuum permeability, saturation magnetization, thickness of the ferromagnetic layer, reduced Planck constant, current density, and H_{DL}/H_{FL}. The details of the data analysis refer to[6-8].

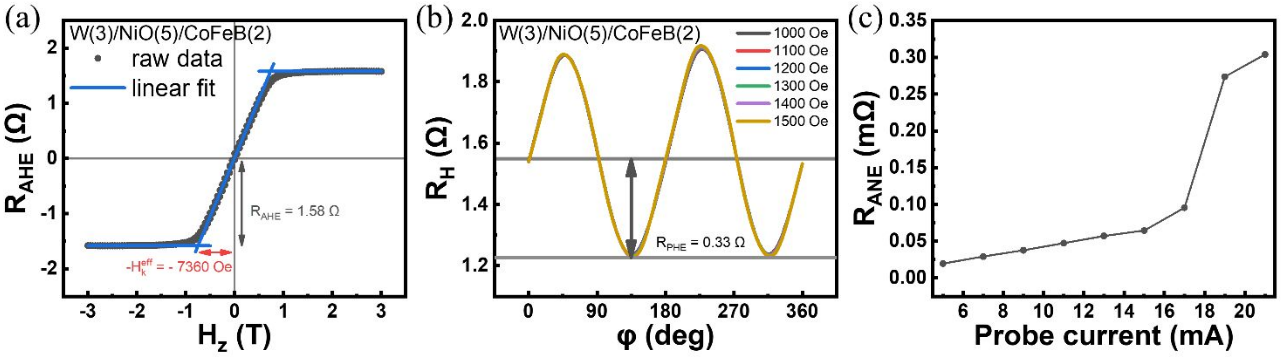

Figure S3 **a**, The Hall effect measurement (R_{AHE}) of W/NiO(5)/CoFeB trilayer taken with a perpendicular field (H_z) reversal. **b**, φ-dependent planar Hall effect (R_{PHE}) of W/NiO(5)/CoFeB trilayer taken with a field rotating in the device plane. **c**, Plots of R_{ANE} signal as a function of the probe current amplitudes acquired via the intercept in the $R_{2\omega}^{DLANE}$ versus $(H_{ext} - H_k^{eff})^{-1}$ correlation.

Supporting information 4 | SOT efficiency characterized using a magnetoresistance-based loop-shift method in y-type geometry

Figure S4-1 exhibits the schematic diagrams to conceptually illustrate the loop-shift method based on y-type geometry, together with the resulting hysteresis curves (MH) and magnetoresistance curves (MR) with varying the probe current amplitudes. Without applying a current through the device, both MH and MR curves appear to be centrosymmetric as presented in **Figure S4-1** (left set). While applying a current, the spin current accumulated at the vicinity of FM would give rise to an effective field at the interface thus biasing the MH and MR curves, as presented in **Figure S4-1** (middle set). Upon further increasing the applied current amplitude, the effective field at the interface gets even stronger and heightens the bias on the MH and MR curves as presented in **Figure S4-1** (right set), which can be quantitatively indicated by the H_{Shift}.

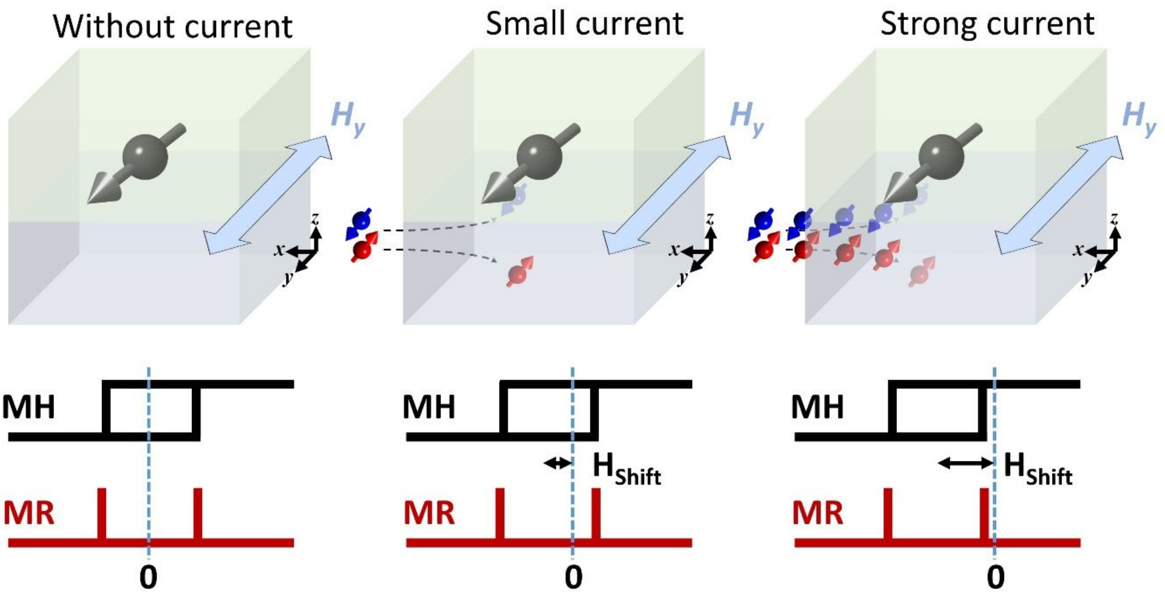

Figure S4-1 Conceptual diagrams to elaborate the fundamentals of the magnetoresistance-based loop-shift method in y-type geometry along with the corresponding hysteresis curve (black) and magnetoresistance (red) curves at the bottom, in which the magnetization and reversal are set in the y-axis. Upon increasing the applied current amplitude, the boosted spin accumulation at the vicinity of FM would magnetically bias the magnetization gradually, thus giving rise to an H_{Shift} on both MH and MR curves.

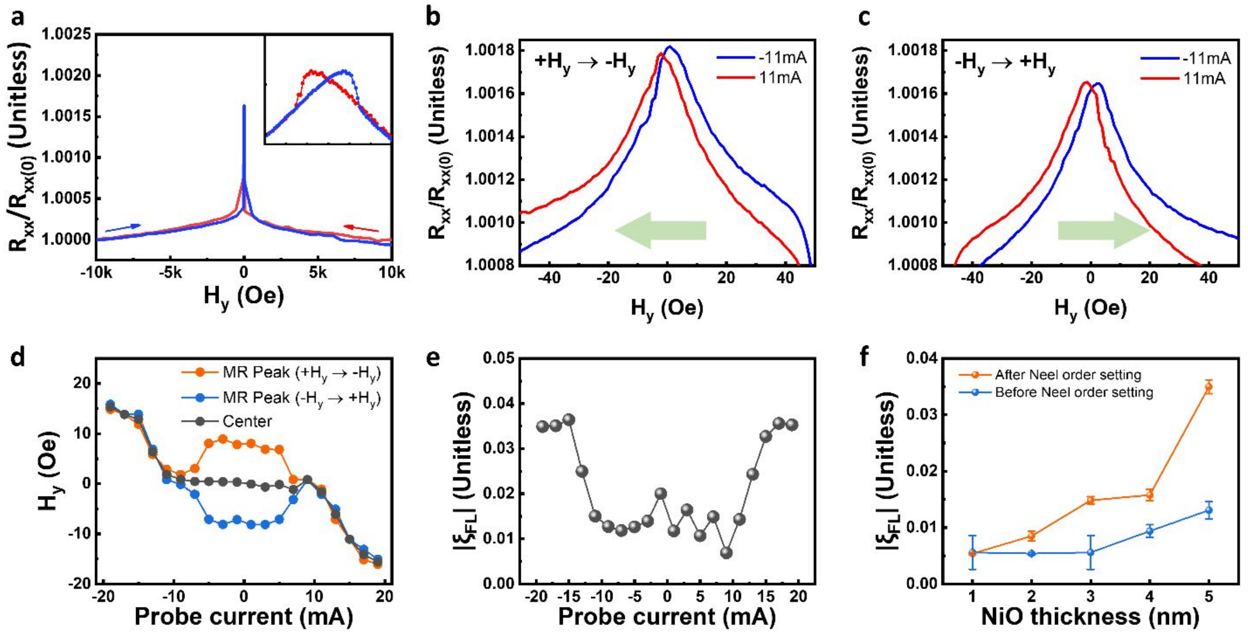

Figure S4-2 **a**, MR curve of the trilayer with NiO(5) taken with a probe current of 1 mA in y-type geometry, where the red (blue) curve represents the MR taken from $+H_y$ ($-H_y$) to $-H_y$ ($+H_y$). Inset: The enlarged MR curve at the central range (-20 Oe ~ +20 Oe) to highlight the two MR maxima. **b**, The highlighted MR peaks taken using $\pm$ 11 mA in the field reversal from $+H_y$ to $-H_y$. **c**, The highlighted MR peaks taken using $\pm$ 11 mA in the field reversal from $-H_y$ to $+H_y$. **d**, Plots of the MR peak position (H_y) obtained from the $+H_y \to -H_y$ (orange) and $-H_y \to +H_y$ (blue), and the center of the two MR peaks (grey) as an indicator of the H_{Shift} to reveal the current induced effective field along y-direction. **e**, The converted field-like torque efficiency (ξ_{FL}) after employing the equation $\xi_{FL} = (\frac{2e\mu_0 M_S t_{FM}}{\hbar})(\frac{H_{Shift}}{J_e})$, where the e, μ_0, M_s, t_{FM}, $\hbar$, and J_e are quantity of electron charge, vacuum permeability, saturation magnetization, thickness of the ferromagnetic layer, reduced Planck constant, and current density, respectively. **f**, Plots for the field-like torque efficiency of the trilayer device with varying NiO thickness taken before and after treating the threshold probe current. The details of the data analysis refer to[6].

Supporting information 5 | SOT efficiency characterizations for Pt/NiO(t)/CoFeB trilayer

Figure S5a and **Figure S5b** show the harmonic Hall measurements and 2nd harmonic Hall signal with φ-dependence in the W/NiO(5)/CoFeB trilayer, respectively, as demonstrated in the Figure 2a and Figure 2c in the main content of the manuscript. **Figure S5c** and **Figure S5d** show the harmonic Hall measurements taken using ±23 mA and 2nd harmonic Hall signal with φ-dependence, respectively, in the Pt/NiO(5)/CoFeB trilayer as a reference. As the result, the phase of 2nd harmonic Hall signal is opposite to that acquired from W/NiO(5)/CoFeB trilayer, agreeing well with the circumstance that Pt and W are the heavy metals with opposite spin Hall angle. **Figure S5e** and **Figure S5f** demonstrate the converted damping-like torque efficiency (ξ_{DL}) and field-like torque efficiency (ξ_{FL}) of the Pt/NiO(t)/CoFeB trilayer as a function of NiO thickness characterized using the harmonic Hall measurement. The evolution on both ξ_{DL} and ξ_{FL} of the Pt/NiO(t)/CoFeB trilayer before and after Néel order setting appears to be in the similar trend as the cases in W/NiO(t)/CoFeB trilayer.

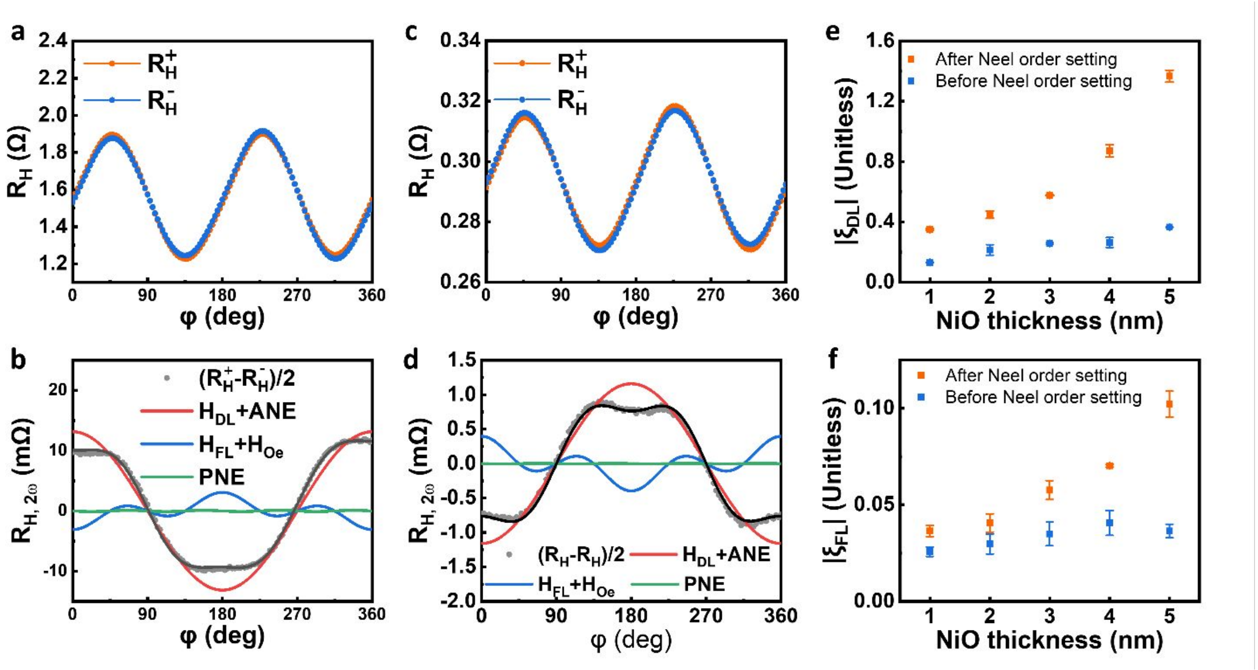

Figure S5 a, φ-dependent harmonic Hall measurements and **b**, 2nd harmonic Hall signal with φ-dependence in the W/NiO(5)/CoFeB trilayer. **c**, φ-dependent harmonic Hall measurements and d, 2nd harmonic Hall signal with φ-dependence in the Pt/NiO(5)/CoFeB trilayer. **e**, Plots of ξ_{DL} and **f**, ξ_{FL} of the Pt/NiO(t)/CoFeB trilayer as a function of NiO thickness.

Supporting information 6 | Geometrical effect of the eight-terminal device on the orthogonal re-writing examination.

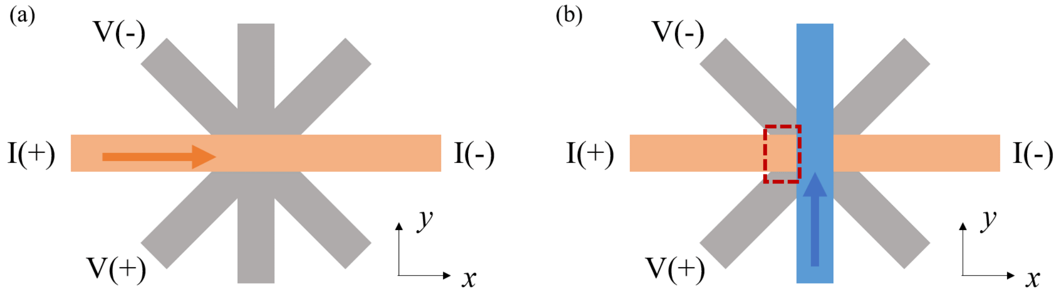

Figure S6 a, Schematic diagram to exhibit the active region (highlighted by orange) of the SOT-driven Néel order switching as indicated by orange after applying the current along x-direction of the device. **b**, Re-writable region (highlighted by blue) of the device after applying the current along y-direction of the device. Red-dashed rectangle in **b** exhibits the unaltered region after the y-direction re-writing, which is responsible for the harmonic Hall measurement.

Figure S6a illustrates the direct writing process by applying current along the x-direction, where the entire current channel is expected to be influenced, thus setting the Néel order as indicated by the orange region. However, when current is applied along the y-direction, as shown in **Figure S6b**, it primarily influences the transverse channel of the device (blue region), reorienting the Néel order only in the central region of the device. As a result, there remains an unaltered area from the y-direction rewriting, which is still responsible for the SOT efficiency as a form of leakage while collecting the harmonic Hall signal through the oblique channels (with a 45° correlation between V(+) and V(-)) of the device. The remnant region explains why the SOT efficiency after y-direction rewriting is still slightly higher than that of the as-grown state. This geometrical effect is almost inevitable in the eight-terminal geometry allowing an orthogonal rewriting functionality.

Reference

1 Chen, X. *et al.* Antidamping-torque-induced switching in biaxial antiferromagnetic insulators. *Physical review letters* **120**, 207204 (2018).

2 Cheng, Y., Yu, S., Zhu, M., Hwang, J. & Yang, F. Electrical switching of tristate antiferromagnetic Néel order in α-Fe 2 O 3 epitaxial films. *Physical Review Letters* **124**, 027202 (2020).

3 Zhang, P. *et al.* Control of Néel vector with spin-orbit torques in an antiferromagnetic insulator with tilted easy plane. *Physical review letters* **129**, 017203 (2022).

4 Peres, N. Spin flop transition in doped antiferromagnets. *Journal of Physics: Condensed Matter* **15**, 7271 (2003).

5 Qian, G. *et al.* Spin-flop transition and Zeeman effect of defect-localized bound states in the antiferromagnetic topological insulator MnBi2Te4. *Nano Research* **16**, 1101-1106 (2023).

6 Liu, Y.-T. *et al.* Determination of spin-orbit-torque efficiencies in heterostructures with in-plane magnetic anisotropy. *Physical Review Applied* **13**, 044032 (2020).

7 Luo, F. *et al.* Simultaneous determination of effective spin-orbit torque fields in magnetic structures with in-plane anisotropy. *Physical Review B* **95**, 174415 (2017).

8 Avci, C. O. *et al.* Interplay of spin-orbit torque and thermoelectric effects in ferromagnet/normal-metal bilayers. *Physical Review B* **90**, 224427 (2014).
